# Aβ-induced vulnerability propagates via the brain’s default mode network

**DOI:** 10.1038/s41467-019-10217-w

**Published:** 2019-06-04

**Authors:** Tharick A. Pascoal, Sulantha Mathotaarachchi, Min Su Kang, Sara Mohaddes, Monica Shin, Ah Yeon Park, Maxime J. Parent, Andrea L. Benedet, Mira Chamoun, Joseph Therriault, Heungsun Hwang, A. Claudio Cuello, Bratislav Misic, Jean-Paul Soucy, John A. D. Aston, Serge Gauthier, Pedro Rosa-Neto

**Affiliations:** 1 Translational Neuroimaging Laboratory, The McGill University Research Centre for Studies in Aging, H4H 1R3 Montreal, Canada; 2 Montreal Neurological Institute, H3A 2B4 Montreal, Canada; 3 Statistical Laboratory, University of Cambridge, CB3 0WB Cambridge, UK; 4 Department of Psychology, McGill University, Montreal, Canada; 5 Department of Pharmacology and Therapeutics, McGill University, H3A 2T5 Montreal, Canada; 6 Alzheimer’s Disease Research Unit, The McGill University Research Centre for Studies in Aging, H4H 1R3 Montreal, Canada

**Keywords:** Positron-emission tomography, Neuroscience, Alzheimer's disease, Dementia

## Abstract

The link between brain amyloid-β (Aβ), metabolism, and dementia symptoms remains a pressing question in Alzheimer’s disease. Here, using positron emission tomography ([^18^F]florbetapir tracer for Aβ and [^18^F]FDG tracer for glucose metabolism) with a novel analytical framework, we found that Aβ aggregation within the brain’s default mode network leads to regional hypometabolism in distant but functionally connected brain regions. Moreover, we found that an interaction between this hypometabolism with overlapping Aβ aggregation is associated with subsequent cognitive decline. These results were also observed in transgenic Aβ rats that do not form neurofibrillary tangles, which support these findings as an independent mechanism of cognitive deterioration. These results suggest a model in which distant Aβ induces regional metabolic vulnerability, whereas the interaction between local Aβ with a vulnerable environment drives the clinical progression of dementia.

## Abstract (web summary)

Amyloid-β (Aβ) deposition occurs in Alzheimer's disease but its relation to disease features such as local brain hypometabolism or cognitive decline is unclear. Here, the authors show that Aβ aggregation in the brain’s default mode network leads to hypometabolism in distant but functionally connected areas.

## Introduction

The relationship between regional cerebral amyloid-β (Aβ) aggregation and hypometabolism has been a topic of significant debate in Alzheimer’s disease (AD). Whereas some studies suggest an association^1–3^, others refute that these pathological processes are directly related^4,5^. The lack of association between these pathologies has been supported by studies, showing the absence of hypometabolism in some brain regions with high Aβ load, as well as the presence of hypometabolism in other areas with low Aβ concentrations^1,6,7^. Notably, the lack of association between regional Aβ and hypometabolism contrasts with the idea of a direct deleterious effect of Aβ on disease progression, as initially proposed in the Aβ hypothesis^8^.

One possible explanation that links Aβ with disease progression and integrates the aforementioned conflicting observations is the idea that regional hypometabolism is caused by the toxic effects of Aβ aggregation from distant rather than topographically overlapping brain regions. Indeed, early observations using [^18^F]fluorodeoxyglucose ([^18^F]FDG) positron emission tomography (PET) support this notion, by showing that brain damage may lead to hypometabolism in remote, but metabolically connected cortical areas^9–11^. These results highlight the possibility of a direct deleterious effect of Aβ on distant hypometabolism, which might be being overlooked by the current biomarker studies.

Regardless of the presence of a regional correlation between Aβ and hypometabolism, it is well established that the topographic coexistence of these pathologies in some brain regions such as the posterior cingulate cortex constitutes a signature of forthcoming dementia symptoms^12–15^. Also, studies have shown that either Aβ or hypometabolism may be the first abnormality in these regions^16,17^, and that cognitive changes may be potentiated by an interaction between these processes^18^. Together, these results indicate that although regional Aβ may not be the cause of its overlapping hypometabolism, progression to dementia may be the result of local interactions between these pathologies.

Here, we tested the hypothesis that distant and local Aβ effects play a distinct role in the progression of AD. We find that distant Aβ determines the region’s metabolic vulnerability, whereas the synergy between this regional vulnerability with co-localized concentrations of Aβ determines dementia. These results were obtained in cognitively normal (CN) and mild cognitive impairment (MCI) individuals as well as transgenic rats that display Aβ single pathology in the absence of other brain human proteinopathies, such as neurofibrillary tangles. We therefore conclude that the deleterious effect of Aβ on the progression of AD occurs in a two-step process, in which distant Aβ determines regional vulnerability and local Aβ drives cognitive decline.

## Results

### Aβ is unrelated to local hypometabolism

Demographics and key characteristics of the human population are summarized in Table 1. Voxel-wise analysis of covariance showed that compared with CN Aβ-negative individuals, CN Aβ positive and MCI Aβ positive had reduced gray matter density in the medial temporal cortex (Supplementary Fig. 1a, d). CN Aβ positive and MCI Aβ positive had reduced metabolism in the precuneus, posterior cingulate, inferior parietal, and temporal cortices (Supplementary Fig. 1b, e), and had widespread Aβ deposition (Supplementary Fig. 1c, f).

**Table 1:** Demographics and key characteristics of the population

	CN Aβ negative	CN Aβ positive	MCI Aβ positive	*P*-values
Number of subjects	99	53	170	–
Age, y, mean (SD)	74.5 (6.7)	75.3 (6.9)	73.2 (6.7)	0.090
Male, no. (%)	56 (57)^b^	15 (28)	88 (52)^b^	0.002
Education, y, mean (SD)	16.5 (2.8)	16 (2.3)	15.9 (2.7)	0.234
APOE ε4, no. (%)	20 (20)	20 (38)^a^	106 (62)^a,b^	<0.001
[^18^F]Florbetapir, mean SUVR (SD)	1.07 (0.05)	1.30 (0.09)^a^	1.35 (0.11)^a,b^	<0.001
MMSE, score, mean (SD)	29.1 (1.2)	28.9 (1.1)	27.7 (1.9)^a,b^	<0.001
MMSE, slope of change, y, mean (SD)	−0.08 (0.52)	−0.12 (0.57)	−0.95 (1.65)^a,b^	<0.001
Follow-up, y, mean (SD)	3.41 (0.97)	3.49 (1.0)	3.26 (1.07)	0.256
Visits, no. (SD)	3.75 (0.69)	3.75 (0.81)	4.06 (1.08)^a^	0.010

*P*-values indicate the values assessed with analyses of variance for each variable, except for gender and APOE ε4 status where a contingency chi-square was performed. Post-hoc analysis provided significant differences between groups from:

^a^CN Aβ negative

^b^CN Aβ positive

**Fig. 1:**
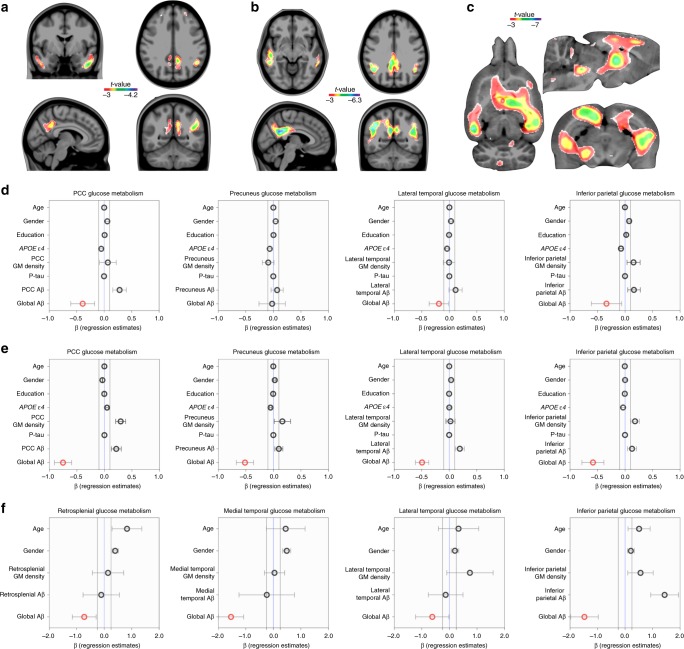
Global rather than local amyloid-β (Aβ) is associated with DMN hypometabolism. Voxel-wise models, taking voxel and global Aβ values into consideration, showed that global Aβ is associated with hypometabolism in (**a**) CN Aβ positive (*n* = 53) and in (**b**) MCI Aβ-positive (*n* = 170) individuals in the posterior cingulate, precuneus, lateral temporal, and inferior parietal cortices, and in (**c**) transgenic Aβ rats (*n* = 10) in the retrosplenial (which corresponds to the posterior cingulate in humans), medial and lateral temporal, and inferior parietal cortices. The effects of regional Aβ values on its overlapping hypometabolism were negligible in the voxel-wise models. Regression models performed in (**d**) CN Aβ-positive and in (**e**) MCI Aβ-positive individuals within anatomically segregated regions further supported that the effects of local Aβ on hypometabolism (i.e., in the same region) were negligible. Similarly, inside segregated clusters, local Aβ effects on hypometabolism were negligible in (**f**) transgenic Aβ rats. In panels **d**–**f**, the dots and bars represent β estimates and standard error, respectively, of the independent variables used in the models. Parametric images were FWER corrected at *P* < 0.05 and adjusted for age, gender, education, APOE ε4 status, p-tau, and gray matter (GM) density in humans, and age, gender, and gray matter density in rats

Voxel-wise models showed that Aβ was not locally associated with hypometabolism in CN Aβ-positive and MCI Aβ-positive individuals. On the other hand, these models showed that global Aβ was associated with hypometabolism in the posterior cingulate, precuneus, lateral temporal, and inferior parietal cortices in CN Aβ positive (Fig. 1a, d; Supplementary Fig. 2a; and Supplementary Fig. 3a) and MCI Aβ positive (Fig. 1b, e; Supplementary Fig. 2b; and Supplementary Fig. 3b). In addition, a bootstrap-scheme supported that regional Aβ did not affect the stability of the global Aβ effects on hypometabolism in the models mentioned above (Supplementary Fig. 4).

We further replicated the results found in humans using a cohort of 20 rats (10 homozygous McGill-R-Thy1-APP rats overexpressing human Aβ precursor protein and 10 wild-type Wistar rats). They were 11-month-old, and the wild-type rats provided the means for determining that 11-month McGill-R-Thy1-APP transgenic rats presented mild cognitive symptoms, with a significant baseline genotype effect on the Morris Water Maze (MWM) (*P* = 0.03). In the transgenic Aβ rats, voxel-wise regressions supported that local Aβ and hypometabolism were unrelated to one another. On the other hand, the model revealed that global Aβ load was strongly associated with hypometabolism in the retrosplenial (which corresponds to the posterior cingulate in humans^19^), medial and lateral temporal, and inferior parietal cortices (Fig. 1c, f).

### Aβ leads to hypometabolism in functionally connected regions

**Fig. 2:**
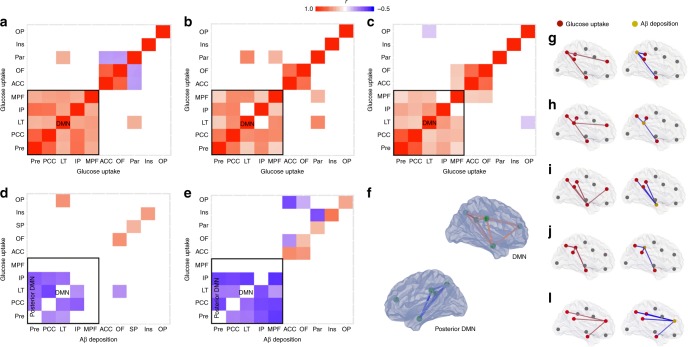
Amyloid-β (Aβ) is associated with hypometabolism in distant, but functionally connected brain regions. Metabolic connectivity analysis between eight regions-of-interest used to compose the global Aβ value in the precuneus (Pre), posterior (PCC) and anterior (ACC) cingulate, inferior parietal (IP), paracentral (Par), medial prefrontal (MPF), lateral temporal (LT), and orbitofrontal (OF), as well as two additional regions in the insular (Ins) and occipital pole (OP) cortices demonstrated that regions comprising the DMN were highly correlated with each other in (**a**) CN Aβ negative (*n* = 99), (**b**) CN Aβ positive (*n* = 53), and (**c**) MCI Aβ positive (*n* = 170) individuals. Partial correlation analysis showed that Aβ within the DMN was associated with distant but within-network hypometabolism in (**d**) CN Aβ-positive and (**e**) MCI Aβ-positive individuals. Note that Aβ and its overlapping metabolism showed positive or non-correlation in these individuals. Correlation maps displayed in 3D brain surfaces show the representations of (**f**) the DMN and posterior DMN and correlations of glucose–glucose (left side) and Aβ-glucose (right side) in the (**g**) Pre, (**h**) PCC, (**i**) LT, (**j**) IP, and (**l**) MPF cortices in MCIs Aβ positive (see Supplementary Movie 1). The matrices are presented with Pearson partial correlation coefficients (*r*) controlled for age, gender, education, *APOE ε4* status, p-tau, gray matter density, and Bonferroni-corrected at *P* < 0.05

In CN Aβ-negative (Fig. 2a), CN Aβ-positive (Fig. 2b), and MCI Aβ-positive (Fig. 2c) individuals, metabolic connectivity analysis showed that the regions comprising the brain's DMN in the precuneus, posterior cingulate, lateral temporal, inferior parietal, and medial prefrontal cortices were highly correlated with each other.

Partial correlation matrices using anatomical segregated regions-of-interest revealed that Aβ was negatively associated with metabolism in distant, but metabolically connected brain regions in CN Aβ-positive (Fig. 2d) and MCI Aβ-positive (Fig. 2e–l; Supplementary Movie 1) individuals. Notably, the elements of the Aβ-glucose and glucose–glucose matrices were highly negatively correlated with one another in these individuals (*r* = −0.5, *P* < 0.0001), which reinforced the link between metabolic connectivity and Aβ effect. Furthermore, the lack of correlation between the Aβ-glucose matrix elements and the Euclidean distance between regions (*r* = 0.1, *P* = 0.3294) supported that regions' proximity did not drive these correlations.

**Fig. 3:**
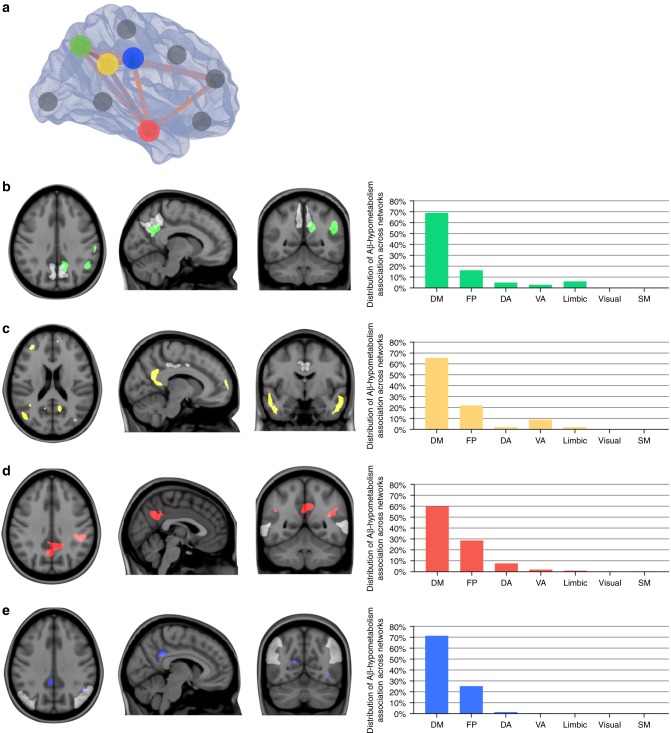
Amyloid-β (Aβ) in DMN is predominantly associated with distant within-network hypometabolism in CN Aβ positive. **a** In the 3D brain, the dots represent the regions-of-interest in which Aβ values were obtained. The regions in which Aβ values were obtained are also shown in white, superimposed on the structural MRI templates in panels **b**–**e**. Statistical parametric maps, overlaid on a structural MRI template, show the brains regions where voxel-wise glucose metabolism was negatively associated with Aβ load in the (**b**) precuneus (green), (**c**) posterior cingulate (yellow), (**d**) lateral temporal (red), and (**e**) inferior parietal (blue) cortices in CN Aβ positive (*n* = 53). The bar graphs show the distribution of the voxels in the aforementioned statistical parametric maps across seven functional brain networks (DM default mode, FP frontoparietal, DA dorsal attention, VA ventral attention, limbic visual, SM somatomotor). Thus, the sum of the seven bars in each graph is 100%. Aβ in the medial prefrontal, anterior cingulate, orbitofrontal, paracentral, insular, and occipital pole cortices (gray dots) did not significantly associate with hypometabolism. Parametric images were FWER corrected at *P* < 0.05 and adjusted for age, gender, education, APOE ε4 status, p-tau, and gray matter density

**Fig. 4:**
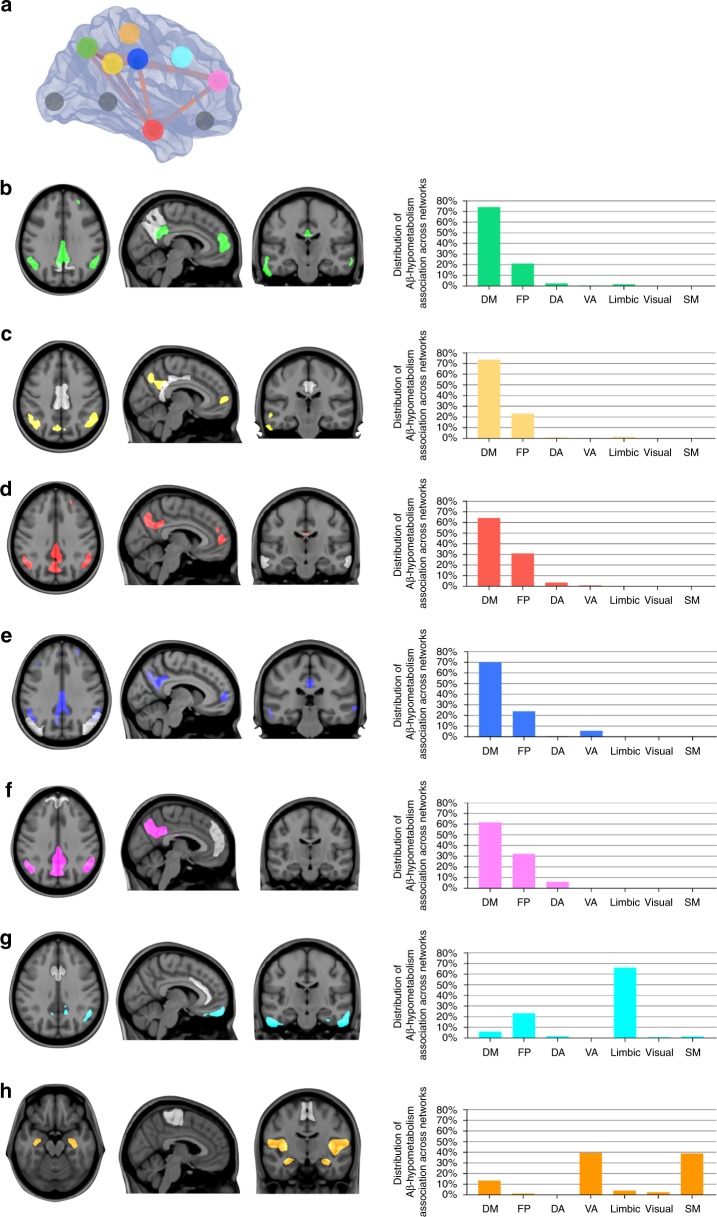
Amyloid-β (Aβ) in DMN is predominantly associated with distant within-network hypometabolism in MCI Aβ positive. **a** In the 3D brain, the dots represent the regions-of-interest in which Aβ values were obtained. The regions in which Aβ values were obtained are also shown in white, superimposed on the structural MRI templates in panels **b**–**h**. Statistical parametric maps, overlaid on a structural MRI template, show the brains regions where voxel-wise glucose metabolism was negatively associated with Aβ load in the (**b**) precuneus (green), (**c**) posterior cingulate (yellow), (**d**) lateral temporal (red), (**e**) inferior parietal (blue), (**f**) medial prefrontal (pink), (**g**) anterior cingulate (light blue), and (**h**) paracentral (orange) cortices in MCI Aβ positive (*n* = 170). The bar graphs show the distribution of the significant voxels in the aforementioned statistical parametric maps across seven functional brain networks (DM default mode, FP frontoparietal, DA dorsal attention, VA ventral attention, limbic visual, SM somatomotor). Thus, the sum of the seven bars in each graph is 100%. Aβ in the orbitofrontal, insular, and occipital pole cortices (gray dots) did not significantly associate with hypometabolism. Parametric images were FWER corrected at *P* < 0.05 and adjusted for age, gender, education, APOE ε4 status, p-tau, and gray matter density

Voxel-wise network analysis showed that Aβ load in the precuneus, posterior cingulate, lateral temporal, inferior parietal, and medial prefrontal cortices were associated with distant, but within-DMN hypometabolism in CN Aβ-positive (Fig. 3) and MCI Aβ-positive (Fig. 4b–f) individuals. On the other hand, Aβ outside of the DMN either did not associate with hypometabolism or was associated with hypometabolism predominantly in regions comprising other brain networks, such as the limbic and ventral attention networks (Fig. 4g, h).

**Fig. 5:**
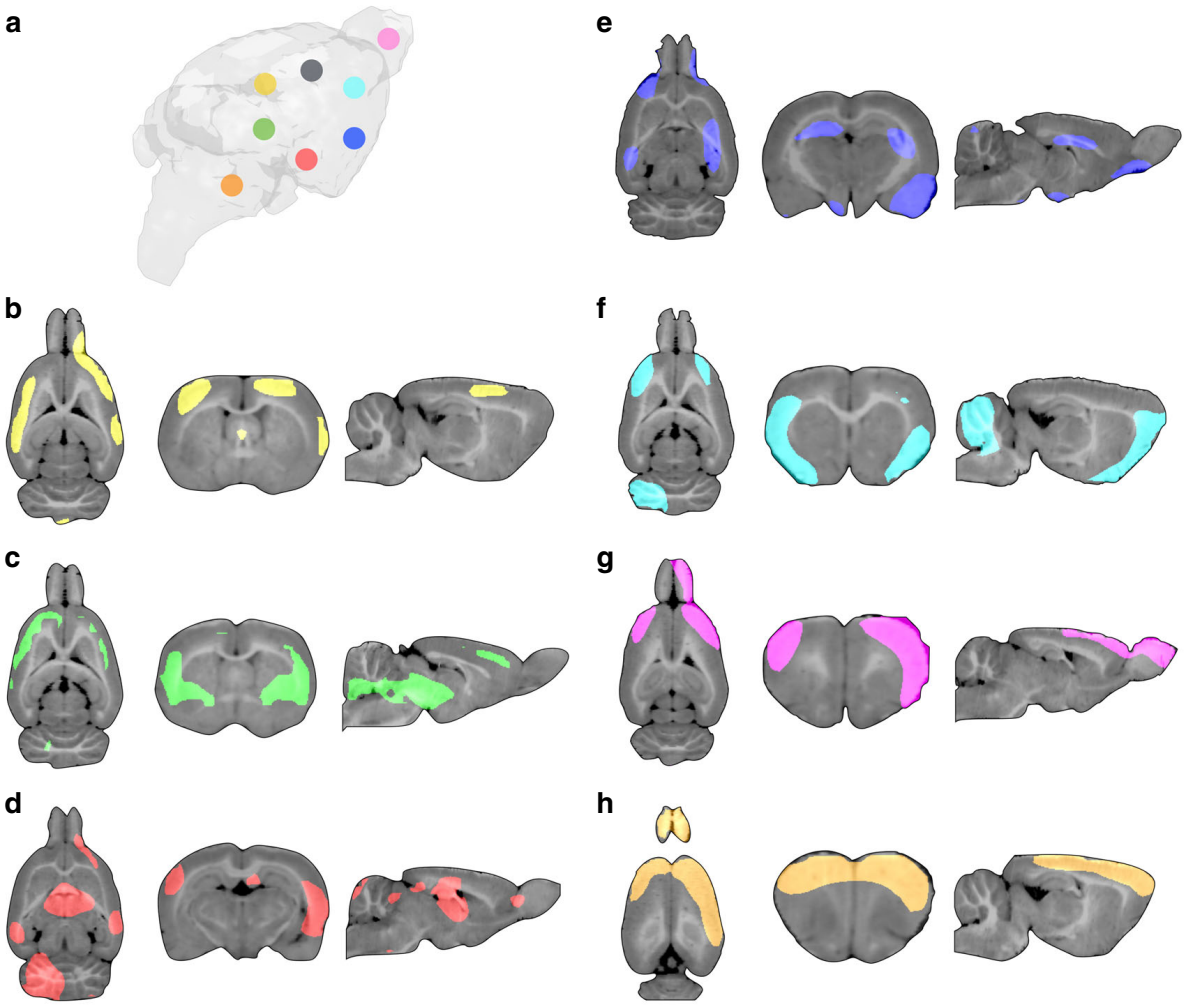
Amyloid-β (Aβ) is associated with distant within-network hypometabolism in transgenic Aβ rats. **a** 3D brain representation of the regions-of-interest in which the Aβ values were obtained. Statistical parametric maps, overlaid on a structural MRI template, show the brains regions where voxel-wise glucose metabolism was negatively associated with Aβ load in the (**b**) retrosplenial (yellow), (**c**) medial temporal (green), (**d**) lateral temporal (red), (**e**) inferior parietal (blue), (**f**) frontoparietal (light blue), (**g**) olfactory bulb (pink), and (**h**) cerebellar (orange) cortices in transgenic Aβ rats (*n* = 10). Aβ in the somatosensory cortex (gray dot) did not significantly associate with hypometabolism. Parametric images were FWER corrected at *P* < 0.05 and adjusted for age, gender, and gray matter density

In the transgenic Aβ rats, a voxel-wise regression analysis confirmed that Aβ was associated with distant, rather than co-localized, hypometabolism (Fig. 5). As expected, there were no associations between Aβ uptake and metabolism in the wild-type control rats.

### Synergy of Aβ and local hypometabolism on cognitive decline

**Fig. 6:**
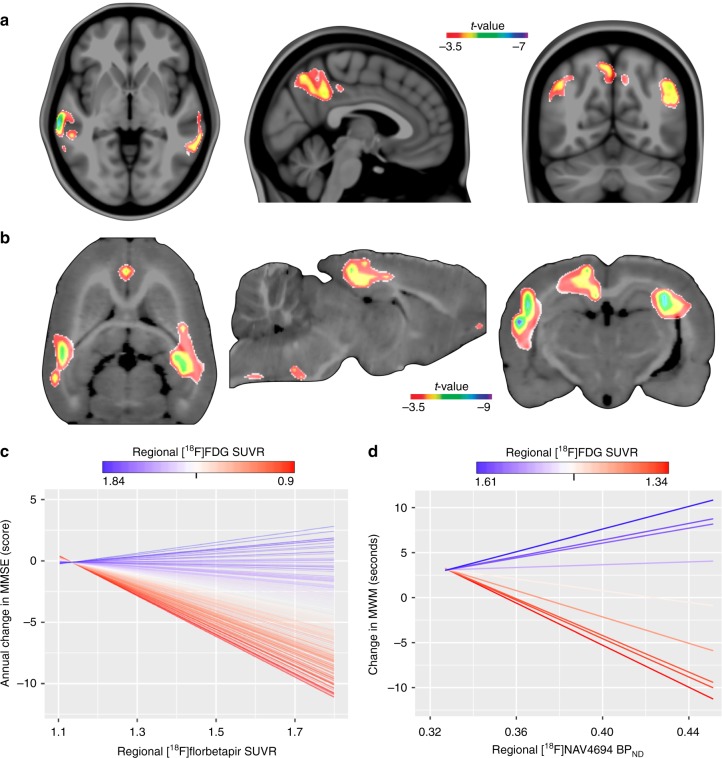
The synergy of amyloid-β (Aβ) with overlapping hypometabolism drives cognitive decline. **a** The parametric map shows significant interactive effects at a voxel level between Aβ and glucose uptake on MMSE worsening over up to 5.6 years in the precuneus, posterior cingulate, inferior parietal, and lateral temporal cortices in MCIs Aβ positive (*n* = 170). The aforementioned interaction was not significantly associated with cognitive decline in CN Aβ positive. **b** In transgenic Aβ rats, significant voxel-wise interactive effects between Aβ and glucose uptake on MWM worsening over 8 months were found in the retrosplenial cortex, which corresponds to the posterior cingulate in humans, inferior parietal, and mediobasal and lateral temporal cortices. The plots show the graphical representation of the interaction in (**c**) MCIs Aβ positive (see Supplementary Movie 2) and in (**d**) transgenic Aβ rats, where each parallel line represents a single subject. For longitudinal changes in MMSE and MWM, lower values indicate greater impairment. Parametric images were FWER corrected at *P* < 0.05 and adjusted for global Aβ, age, gender, education, *APOE ε4* status, p-tau, gray matter density, and follow-up duration in humans, and adjusted for global Aβ, age, gender, and gray matter density in rats

A voxel-wise interaction model showed that high local levels of Aβ and low local levels of glucose uptake in the precuneus, posterior cingulate, inferior parietal, and lateral temporal cortices synergistically determined subsequent cognitive decline up to 5.6 years in MCI Aβ-positive individuals (Fig. 6a, c; Supplementary Fig. 5; Supplementary Movie 2). In addition, a bootstrap analysis supported that the effect of the local interaction between Aβ and metabolism on cognitive decline was not influenced by a global Aβ effect (Supplementary Fig. 6). Moreover, analysis of variance reinforced that the models with the interaction term best described the relationship between overlapping biomarkers and cognitive decline as compared with reduced models assessing: (1) only Aβ, (2) only metabolism, and (3) Aβ plus metabolism, with a *P* < 0.0001 in all three cases. In CN Aβ-positive individuals, the aforementioned interaction was not significantly associated with cognitive decline.

In the transgenic Aβ rats, a voxel-wise interaction model supported the synergy between Aβ and overlapping hypometabolism in the retrosplenial, inferior parietal, and mediolateral temporal cortices as a determinant of cognitive decline over 8 months (Fig. 6b, d). This interaction was absent in the wild-type control rats.

### Distant and local Aβ effects on hypometabolism

**Fig. 7:**
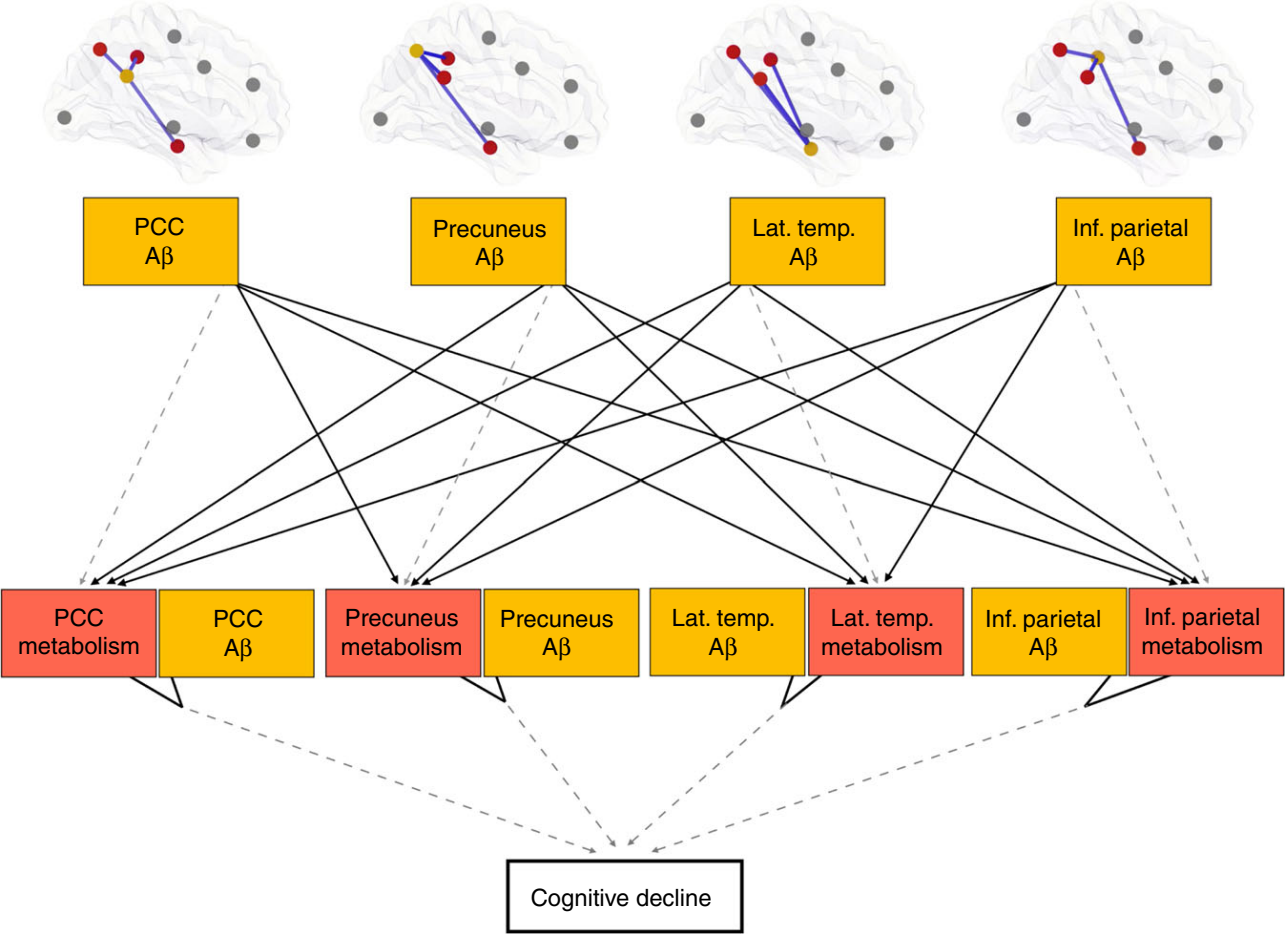
Structured equation modeling showed that the distant and local amyloid-β (Aβ) effects on hypometabolism well describe AD progression. This model represents the hypothesis that distant and local Aβ effects (yellow) on posterior DMN hypometabolism (red) are associated with cognitive decline. The model used CN Aβ-positive and MCI Aβ-positive individuals, and the associations were adjusted for age, gender, education, APOE ε4 status, p-tau, and gray matter density. Negative associations are shown in solid lines, whereas dashed lines show positive associations. Notably, Aβ showed positive association with its overlapping metabolism but negative associations with distant metabolism. The hypothesized model fitted the data well (*n* = 223, *X*^2^ = 81, degrees of freedom = 21, *P* < 0.01, standardized root mean square residual (SRMR) = 0.092, and Comparative Fit Index (CFI) = 0.966)

Structured equation modeling (SEM) showed that the construct in which Aβ occurring in distant regions drives hypometabolism, whereas the interaction between this hypometabolism and the co-localized Aβ determines cognitive decline well describe AD progression (Fig. 7).

## Discussion

**Fig. 8:**
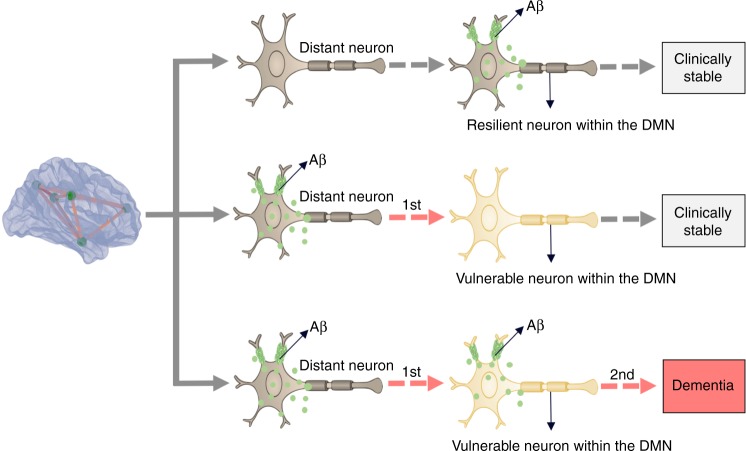
Schematic representation of the distant and local amyloid-β (Aβ) effects on metabolism. Aβ from distant brain regions leads to regional metabolic vulnerability, whereas the synergy between this vulnerability with local Aβ effects drives the clinical progression of dementia. Importantly, either Aβ or metabolic vulnerability as a single abnormality is insufficient to determine dementia

In summary, our results suggest that Aβ is related to vulnerability in distant brain regions functionally connected by the DMN, whereas the synergy between this vulnerability with local Aβ levels is associated with the clinical progression to dementia (Fig. 8). Remarkably, similar results in transgenic Aβ rats, which do not form neurofibrillary tangles^20^, supported this model as an independent mechanism of cognitive deterioration.

Our results suggest that the regional hypometabolism observed in AD may derive from distant Aβ effects within brain regions connected by the DMN. This observation is supported by early studies showing that lesions in remote brain regions are associated with DMN hypometabolism^9–11^. For instance, studies in non-human primates show that localized surgical lesions lead to hypometabolism in metabolically connected brain areas, and that the severity of the inflicted lesion correlates with the degree of remote hypometabolism^11^. These studies suggest that this occurs due to a synaptic disconnection between the damaged and the remote brain region^11^. Based on these observations, one may argue that the regional patterns of hypometabolism in AD derive from a decreased synaptic input from distant, but DMN connected brain areas affected by Aβ. Also, we have shown that global Aβ load, rather than local, is associated with regional DMN hypometabolism, which is in line with previous studies^4^. Expanding upon these studies, our findings indicate that this might be explained by the fact that Aβ from several distant brain regions contributes to regional hypometabolism. Therefore, it seems reasonable that a global Aβ composite contemplating all distant brain regions shows a better association with regional hypometabolism than merely local Aβ levels. Altogether, our results corroborate the deleterious effects of Aβ on the DMN as previously suggested by the network neurodegeneration hypothesis of AD^15,21,22^, by showing that Aβ leads to hypometabolism in distant brain regions interconnected by the DMN, rather than locally, as inferred from the traditional view^22^.

On the other hand, our results support the synergy between Aβ and topographically overlapping DMN vulnerability as a driving force behind dementia symptoms. It is well known that individuals might respond differently to Aβ based on their capacity to compensate through the reconfiguration of pre-existent or recruitment of alternative synapses using the DMN^22,23^. Moreover, DMN hypometabolism has been repeatedly suggested to represent underlying network dysfunction^4,14,24^, and metabolic connectivity has been shown to represent synaptic pathways in the human brain^25–27^. Thus, the hypometabolism in our results may indicate the dysfunction of the DMN in recruiting alternative synapses in the face of toxic effects of Aβ. As such, our model could be understood to mean that the toxic effects of Aβ and the underlying local levels of DMN dysfunction in handling these toxic effects synergistically determine the severity of the subsequent cognitive deterioration. Together, these findings support that regional vulnerability to Aβ plays an important role in the progression to AD and that this occurs through the decrease of the capacity of the DMN to compensate for the deleterious effects of Aβ^28–30^.

Although our results suggested that regional metabolic vulnerability depends on distant Aβ, other factors may potentiate this vulnerability in AD. Determinants of such vulnerability might include inflammatory agents and pathology related stressors that might lead to biochemical dysregulations, resulting in decreased synaptic exchanges and therefore hypometabolism^29,31–33^. Other factors may include cerebral vascular diseases, systemic metabolic disorders, and allele variants implicated in brain plasticity and resilience^34–36^. Also, previous studies have proposed that *APOE ε4* may lead to hypometabolism independently of Aβ^37,38^. Recently, it has been shown that astrocytes play an important role in [^18^F]FDG signal^39^. Since astrocytes have been associated with numerous mechanisms involving neuronal plasticity and transmission^40^, astrocytic dysfunction may be involved in the DMN vulnerability reported here.

As previously reported by our group and others, interactions between Aβ and tau proteins determine AD progression^34,41,42^. Since glucose metabolism is proposed to be closely linked to tau pathology^36^, the regional effects of hypometabolism in this study could be merely inferred as a proxy of neurofibrillary tangles. Therefore, we used the McGill-R-Thy1-APP rats overexpressing human Aβ pathology to clarify our results. Remarkably, the similar results in our transgenic Aβ rats, which do not form tangles^20^, supported the associations between Aβ and DMN vulnerability as a tau-independent mechanism of cognitive deterioration. It is worth to mention that, although independent, this model is likely to be highly potentiated by neurofibrillary tangles, which is supported by the more markedly cognitive deterioration found in humans as compared with the transgenic Aβ rats. Indeed, it is probable that the Aβ-induced metabolic vulnerability reported here will create a favorable environment, also for neurofibrillary tangles to determine cognitive decline. Importantly, the DMN is an evolutionarily conserved feature involved in the integration of cognitive abilities in humans and rodents^19,43^.

Methodological strengths of this study include the use of continuous biomarkers analyzed with a robust voxel-wise approach and the use of an animal model in a controlled experimental setting, which permitted assessing the effects of Aβ on metabolism independent of possible confounding factors, such as neurofibrillary tangles. This study has methodological limitations. Our studied population represents a self-selected group of elderly individuals motivated to participate in an AD study. Therefore, these individuals may not represent a general elderly population. Importantly, the model proposed in this study should be confirmed by future studies using different populations and long-term sequential biomarkers and clinical observations performed at multiple time points. In a heterogeneous population such as MCIs, some individuals have early Aβ deposition and may present a positive association between Aβ and metabolism^44^, while others in later disease stages may present a negative association. Thus, these opposing phenomena in the same population may obscure the regional association between these biomarkers. Although we restricted our analyses to Aβ-positive individuals and adjusted the models for p-tau levels, we cannot exclude that other pathological processes than Aβ—such as tau aggregates, neuroinflammation, cholinergic depletion, cerebrovascular disease, α-synuclein, and TDP-43—have influenced the interpretation of these results^45^. Also, the lack of tau PET images is an important limitation of these results. McGill-R-Thy1-APP rats develop Aβ plaque and oligomeric species^46^. Since regional Aβ load is highly associated with regional oligomeric Aβ^32^, the deleterious effects of Aβ on metabolism reported here might reflect oligomers rather than fibrillar conformations. Also, there are large pathophysiological differences between human AD and transgenic models overexpressing Aβ. However, it is important to mention that the less aggressive progression of Aβ in rats, compared with mice, makes this model more similar to the insidious disease progression of elderlies with sporadic AD. For instance, McGill-R-Thy1-APP mice and rats express exactly the same mutations, but rats present plaques at 6–9 months while mice do as early as at 4 months^20^. Importantly, the much larger brain size of rats (approximately five times bigger than mice) makes the identification of specific brain structures using techniques with low spatial resolution, such as PET, possible to be performed in this study^47^.

To conclude, our results suggest that within the brain’s DMN, regional vulnerability is associated with distant Aβ, while the interaction between this vulnerability with local Aβ is associated with dementia.

## Methods

### Human subjects

The data used in the preparation of this article were obtained from the Alzheimer's Disease Neuroimaging Initiative (ADNI) database, phases GO, and 2 (adni.loni.usc.edu). ADNI was launched in 2003 as a public-private partnership, led by Principal Investigator Michael W. Weiner, MD. The primary goal of ADNI has been to test whether serial magnetic resonance imaging (MRI), PET, other biological markers, and clinical and neuropsychological assessments can be combined to measure the progression of MCI and early AD. ADNI study was conducted according to Good Clinical Practice guidelines, US 21CFR Part 50—Protection of Human Subjects, and Part 56—Institutional Review Boards, and pursuant to state and federal HIPAA regulations and was approved by the Institutional Review Board of each participating site (adni.loni.usc.edu). Written informed consent for the study was obtained from all participants and/or authorized representatives. We studied participants who had [^18^F]FDG PET, [^18^F]florbetapir PET, MRI, and cerebrospinal fluid (CSF) p-tau at baseline, as well as cognitive assessments at baseline and follow-up. Cognition was assessed with Mini-Mental State Examination (MMSE), Rey Auditory Verbal Learning Test 30-min delayed recall, Trail Making Test Part A and B, and Boston Naming Test. For this study, we selected CN individuals and MCI Aβ-positive individuals. CN individuals had an MMSE score of 24 or higher, and a clinical dementia rating (CDR) of 0. MCIs had MMSE scores equal to or greater than 24, a CDR of 0.5, subjective and objective memory impairments, essentially normal activities of daily living, and were not demented (National Institute of Neurological and Communicative Disorders and Stroke–Alzheimer’s Disease and Related Disorders Association criteria for probable AD^48^). Individuals did not present other neuropsychiatric disorders (further information about ADNI cognitive tests and inclusion/exclusion criteria may be found at www.adni-info.org).

### Animal use

We imaged and cognitively assessed 20 rats, 10 homozygous McGill-R-Thy1-APP rats overexpressing human Aβ precursor protein^20^ with the Swedish double mutation (K670N, M671L^49^) and the Indiana mutation (V717F^50^), along with 10 wild-type Wistar rats. Half of the rats in each group were males. The rats performed MRI, [^18^F]FDG PET, and [^18^F]NAV4694 PET at baseline (11-month-old (SD 0.14)), as well as the MWM test at baseline and at 8 months follow-up. The MWM was performed with four trials per day, over 4 consecutive days. The rats were placed on the platform if they failed to find it within 90  s, and we assumed a maximum trial length of 90 s^51^. The time to find the platform was measured with overhead camera tracking using ANY-maze software (*Stoelting Co*.). One probe trial (no platform) and one visible platform trial were also conducted at the end of the 4th day. All rats were kept in ventilated cages in groups of two in environmentally controlled conditions: 12 h light/dark cycle at 21 °C with access to food and water ad libitum. All procedures involving rats were performed in accordance with the ethical regulations for animal testing and research from the Canadian Council on Animal Care and the National Institutes of Health and received ethical approval from the Animal Care Ethics Committee of the McGill University.

### Human imaging methods

MRI and PET acquisitions followed ADNI protocols (http://adni.loni.usc.edu/methods). T1-weighted MRIs were corrected for field distortions, non-uniformity corrected, brain masked, segmented, and non-linearly transformed to the MNI reference space using the CIVET pipeline^52^. Subsequently, gray matter density was computed using voxel-wise morphometry. PET scans used [^18^F]florbetapir tracer for imaging Aβ and [^18^F]FDG tracer for imaging glucose metabolism. Briefly, PET images were non-linearly registered to the MNI space using the individual’s PET/T1-weighted native MRI registration and the individual’s non-linear MRI transformation to MNI space. PET images were spatially smoothed to achieve a final 8-mm full-width at half-maximum resolution and corrected for partial volume effects using region-based voxel-wise (RBV) method^53^. [^18^F]Florbetapir and [^18^F]FDG SUVR images were obtained using the cerebellum gray matter and the pons as reference regions, respectively. A global [^18^F]florbetapir SUVR value was obtained using the precuneus, posterior and anterior cingulate, inferior parietal, paracentral, medial prefrontal, lateral temporal, and orbitofrontal cortices. Individuals were Aβ positive if [^18^F]florbetapir SUVR > 1.15^54^. Further information and the schematic representation of the human imaging methods pipeline may be found elsewhere^34^ and in the Supplementary Fig. 7.

### Rat imaging methods

MRIs were acquired using a Bruker 7T BioSpec 70/30 USR animal dedicated. First, the rats were anesthetized with 5% isoflurane/medical air, which was then maintained at 1–2% after placing the animals in the scanner, with the breathing at 20–30 breaths per min. We used a 37 °C constant airflow to maintain the rats warm. The structural images were generated using the 7T Bruker standard 3D-True Fast Imaging with Steady State Precession (FISP) pulse sequence^55^. Gray matter density was computed using voxel-wise morphometry. PET images were acquired using a CTI Concorde R4 microPET for rodents (Siemens). PET scans used [^18^F]NAV4694 tracer for imaging Aβ and [^18^F]FDG tracer for imaging glucose metabolism. In preparation for the scans, rats received anesthesia using 5% isoflurane, which was then maintained at 2% throughout the procedure. The animals underwent a 9-min transmission scan using a rotating^57^ Co point source before each PET image acquisition. For [^18^F]NAV4694, a 60-min dynamic emission scan began concomitantly with the bolus injection of the radiotracer via the tail vein. For [^18^F]FDG, after fasting for 12 h, the animals received the radiotracer injection during the awake state via the tail vein. Then, a 20-min dynamic emission scan at 50 min post injection was performed. PET images were reconstructed using a maximum a posteriori algorithm and corrected for scattering, dead time, and decay. [^18^F]NAV4694 non-displaceable binding potential (BP_ND_) parametric images were generated using the Simplified Reference Tissue Method with the cerebellar gray matter as a reference^56^. [^18^F]FDG SUVR images were generated using the pons as the reference region. PET images were co-registered to the animal’s MRI and non-linearly transformed to a standardized rat brain space created based on the wild-type rats MRIs. PET images were spatially smoothed using a Gaussian kernel with a full-width at half-maximum of 2.4 mm. Further information and the schematic representation of the rat imaging methods pipeline may be found elsewhere^47^ and in Supplementary Fig. 8.

### Human CSF analysis

CSF p-tau at threonine 181 was measured using a multiplex xMAP Luminex platform (Luminex Corp, Austin, TX) with INNO-BIA AlzBio3 immunoassay kit-based reagents (Innogenetics, Ghent, Belgium)^57,58^. Details about CSF acquisition and quantification can be found at www.adni-info.org.

### Statistical analysis

**Fig. 9:**
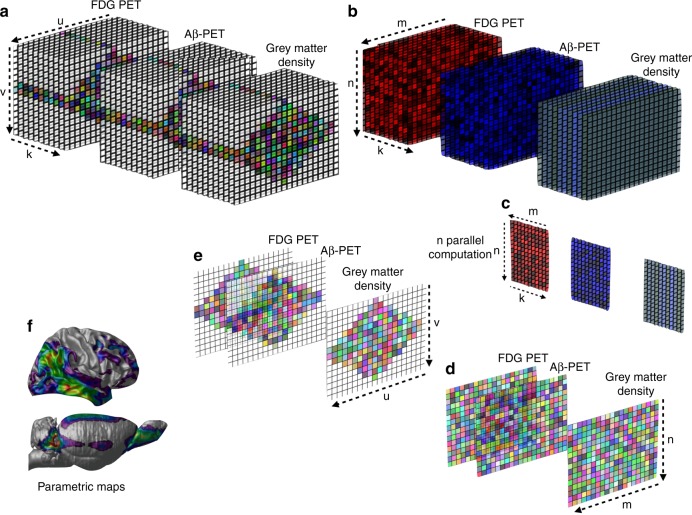
Multimodal analytical operations performed at every brain voxel. The illustration shows the analytical model developed to perform statistical operations on multiple scalar variables and with multiple imaging modalities at every brain voxel in humans and rats. Briefly, (**a**) the brain image data were retrieved from a 3D image space and converted to a 2D matrix in the image space for each subject. **b** Then, the image data were transformed into the processing space using artificial parcellation. **c** In the computational phase, the statistical modeling was performed in every brain voxel accounting for voxel and global PET uptake values, as well as voxel gray matter density and covariates. **d** Subsequently, statistical matrices were generated from the results and (**e**) transformed back to the 3D image space. **f** Finally, 3D parametric maps displaying the results of the regression models were generated. k = subjects, u = image slice, v = slice elements, m = artificial parcellation, *n* = elements in each parcellation

Regressions were performed using R Statistical Software Package version 3.1.2 (http://www.r-project.org/) to test for significant associations between biomarkers and also demographic differences between groups for continuous variables, whereas chi-square was used for categorical variables. The voxel-wise analyses were performed using MATLAB software version 9.2 (http://www.mathworks.com) with a computational framework developed to perform complex voxel-wise statistical operations, such as interaction models, using multiple imaging modalities in humans and rats (Fig. 9)^59^.

In humans, we evaluated the brain abnormalities associated with the clinical diagnosis using a voxel-wise analysis of variance, in which the signal intensity of the imaging modality was used as the dependent variable, while the diagnosis was used as the independent variable.

Subsequently, voxel-wise regression models, taking into consideration voxel and global Aβ concentrations, tested the association between Aβ deposition and glucose metabolism in humans and rats. In humans, we also considered age, gender, education, APOE *ε4* status, p-tau, and gray matter density in the models, whereas in rats, age, gender, and gray matter density were considered.

Then, connectivity analysis was performed using eight regions-of-interest used to assess the global PET Aβ value in the precuneus, posterior and anterior cingulate, inferior parietal, paracentral, medial prefrontal, lateral temporal, and orbitofrontal cortices as well as two additional regions in the insular and occipital cortices, using coordinates in the MNI ICBM atlas^60^. The edge values were assumed to be the correlation coefficients between regions, which were used as the matrix elements. Interregional correlation coefficients were computed using Pearson partial correlation analysis controlling for age, gender, education, *APOE ε4* status, p-tau, and gray matter density. Metabolic connectivity was assessed with a symmetric matrix showing the strength of the correlation of glucose uptake between regions. An asymmetric matrix assessed the associations of Aβ deposition and hypometabolism between regions-of-interest. The matrices were corrected for multiple comparisons using Bonferroni at *P* < 0.05. FDG-FDG and FDG-Aβ matrix elements were further correlated using Pearson's correlation. We also tested the correlation between the matrix elements and the Euclidean distance between nodes (mm) to ensure that the results were not driven by regions' proximity.

In humans, we further performed a voxel-wise connectivity analysis using Aβ values within the 10 regions-of-interest mentioned above as independent predictors and glucose metabolism at every voxel as the outcome accounting for age, gender, education, *APOE ε4* status, p-tau, and gray matter density. In addition, a brain network atlas provided the means to assess the overlap between the results and functional networks (default mode, frontoparietal, dorsal and ventral attention, limbic, visual, and somatomotor networks^61^). In rats, a voxel-wise connectivity analysis was performed using Aβ values in eight regions-of-interest, based on previous literature, in retrosplenial, medial temporal, lateral temporal, inferior parietal, frontoparietal, olfactory bulb, cerebellar, and somatosensory cortices as independent predictors and glucose metabolism at every voxel as the outcome accounting for age, gender, and gray matter density^19,43^.

The association between image biomarkers and cognitive changes was tested in humans with a voxel-wise model using the slope of change in cognitive performance as the dependent variable and the main and interactive effects of [^18^F]florbetapir SUVR and [^18^F]FDG SUVR at every voxel as independent predictors. The slope of change in cognitive performance was defined using all available MMSE evaluations for each subject with a mean of 4 (SD: 0.96) evaluations spanning up to 5.6 years (mean of 3.4 years (SD: 1.02)). The analysis was adjusted for global Aβ, age, gender, education, *APOE ε4* status, p-tau, gray matter density, and follow-up duration. The voxel-wise interaction model was built as follows:

1 $$\begin{array}{l}{\mathrm{\Delta MMSE}} = \beta 0 + \beta 1({\mathrm{Florbetapir}}\,{\mathrm{SUVR}})\\ + \, \beta 2({\mathrm{FDG}}\,{\mathrm{SUVR}})\\ + \, \beta 3({\mathrm{Florbetapir}}\,{\mathrm{SUVR}} \ast {\mathrm{FDG}}\,{\mathrm{SUVR}})\\ + \, {\mathrm{covariates}} + {\mathrm{error}}\end{array}$$

In rats, we built the same voxel-wise model performed in humans using the slope of change in the MWM as the dependent variable and the main and interactive effects of [^18^F]NAV4694 BP_ND_ and [^18^F]FDG SUVR as independent predictors. A slope of change in cognitive performance was defined for each rat using the MWM changes in average (four trials) latency to find the platform over 8 months. The model was adjusted for global Aβ, age, gender, and gray matter density. The voxel-wise interaction model was built as follows:

2 $$\begin{array}{l}{\mathrm{\Delta MWM}} = \beta 0 + \beta 1({\mathrm{NAV}}4694\,{\mathrm{BP}}_{{\mathrm{ND}}})\\ + \, \beta 2({\mathrm{FDG}}\,{\mathrm{SUVR}})\\ + \, \beta 3({\mathrm{NAV}}4694\,{\mathrm{BP}}_{{\mathrm{ND}}} \ast {\mathrm{FDG}}\,{\mathrm{SUVR}})\\ + \, {\mathrm{covariates}} + {\mathrm{error}}\end{array}$$

Statistical parametric maps were corrected for multiple comparisons, and the statistical significance was defined using a family-wise error rate (FWER) threshold of *P* < 0.05.

The presence of collinearity between regional and global Aβ PET values could inflate the variance of estimates, potentially leading to incorrect the statistical results of significance when both effects are used in the same model. To assess whether such case occurred in our analysis, we investigated the stability of estimates by measuring the variance based on a resampling scheme repeated 10,000 times. In addition, the adequacy of the models with the interaction term was further assessed with an analysis of variance comparing the interaction model with each reduced model: (1) Aβ, (2) metabolism, and (3) Aβ plus metabolism.

The relationship between Aβ, metabolism, and the longitudinal cognitive decline was further assessed via SEM using the R package Lavaan^62^. SEM was built to test the specific hypotheses demonstrated in the figure’s meta-model^63^. SEM was classified as satisfactory whether: comparative fit index (CFI) > 0.95 and standardized root mean-square residual (SRMR) < 0.1^64^.

## Supplementary information

Supplementary Information

Supplementary Movie 1

Supplementary Movie 2

## Data Availability

All human data used in this study were downloaded online at the Alzheimer’s Disease Neuroimaging Initiative database (adni.loni.usc.edu). The locally processed human brain images (MRI and PET) and the animal data are not publicly available for download, but might be retrieved from the correspondent author on a request.
