# Supplementary material for: Aβ-induced vulnerability propagates via the brain’s default mode network
Source: Nat Commun. 2019 Jun 4;10:2353. doi: 10.1038/s41467-019-10217-w (PMC6547716; doi:10.1038/s41467-019-10217-w)
Supplement: Supplementary file 1 — Supplementary Information [file 41467_2019_10217_MOESM1_ESM.pdf]

Supplementary Information

Aβ-induced vulnerability propagates via the brain's default mode network

Pascoal *et al.*

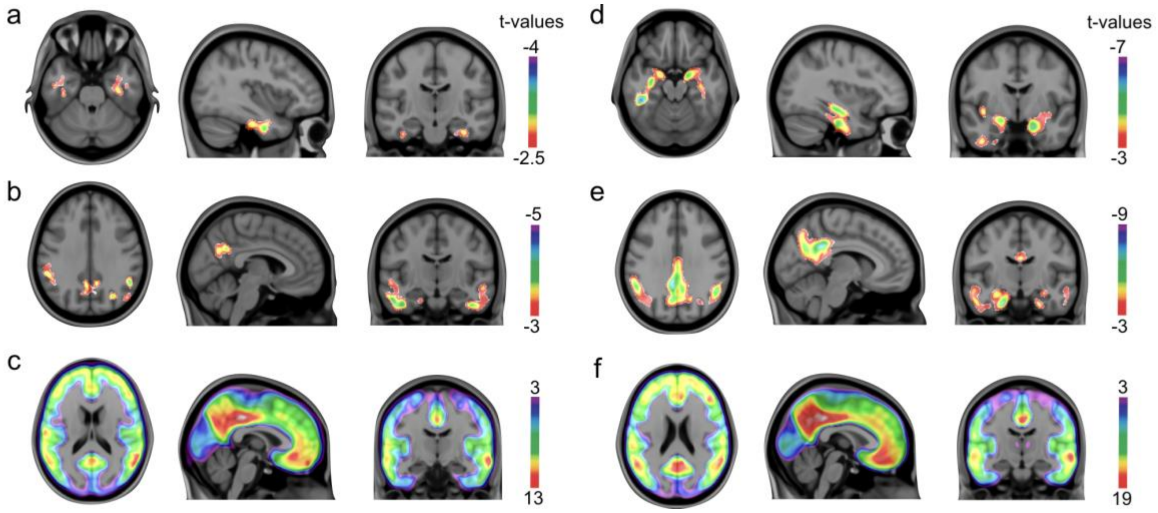

Supplementary Figure 1. Local diagnostic effect on gray matter density, glucose metabolism, and Aβ deposition. Statistical parametric maps, FWER corrected at $P < 0.05$, revealed areas with reduced gray matter density in (**a**) CN Aβ positive ($n = 53$) and (**d**) MCI Aβ positive ($n = 170$); hypometabolism in (**b**) CN Aβ positive and (**e**) MCI CN Aβ positive; and areas with increased Aβ deposition in (**c**) CN Aβ positive and (**f**) MCI Aβ positive compared to CN Aβ negative ($n = 99$) individuals.

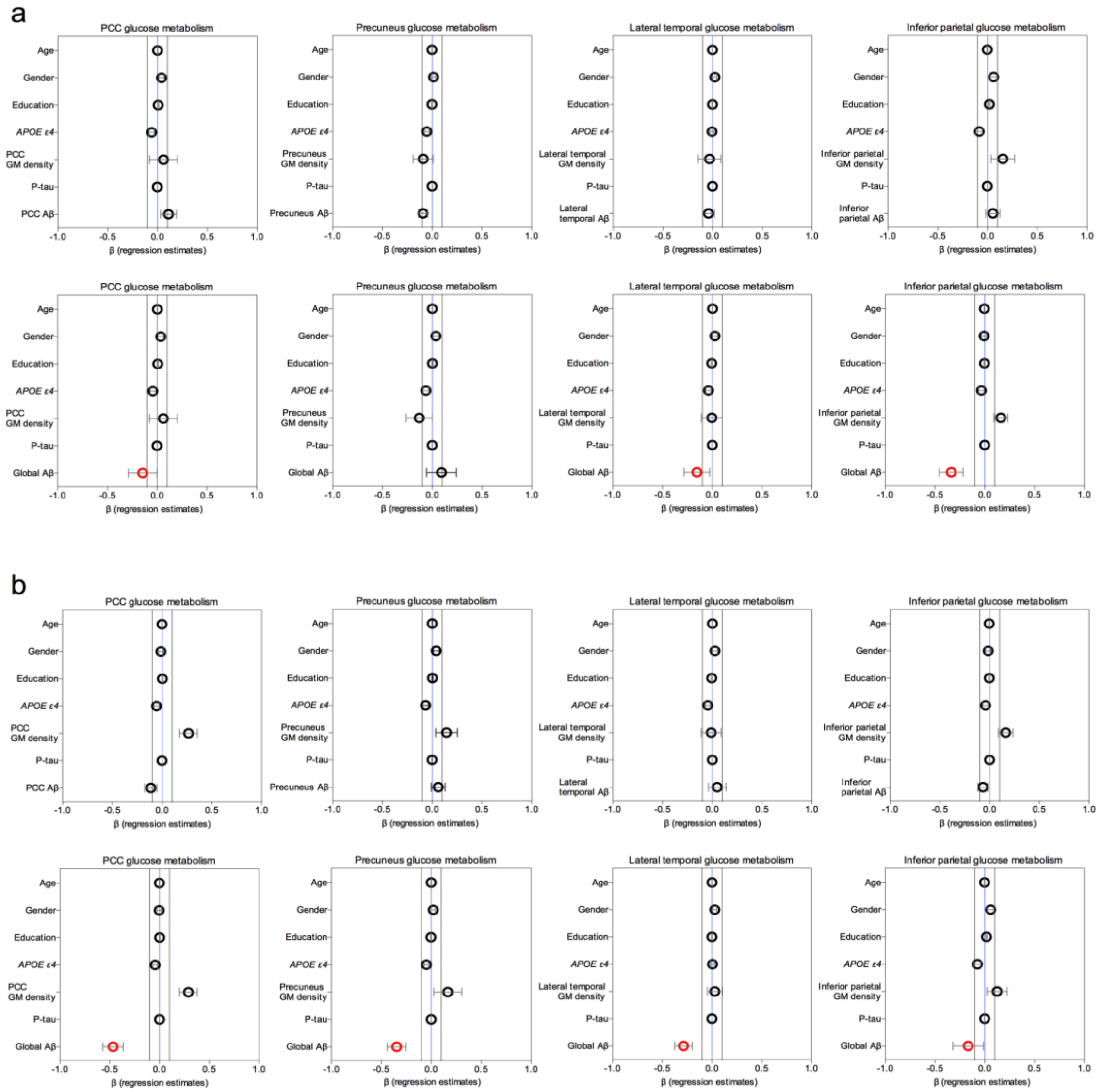

Supplementary Figure 2. Local Aβ was not associated with regional metabolism in CN Aβ positive and MCI Aβ positive individuals. Regression models performed in (**a**) CN Aβ positive (n = 53) and (**b**) MCI Aβ positive (n = 170) individuals within anatomically segregated regions showed that the effects of local Aβ on hypometabolism were negligible even in models without global Aβ. Also, global Aβ was associated with regional metabolism independently of the presence of regional Aβ in the models (see complete models in Fig. 1d, e). In panels a, b the dots and bars represent β estimates and standard error, respectively, of the independent variables used in the models.

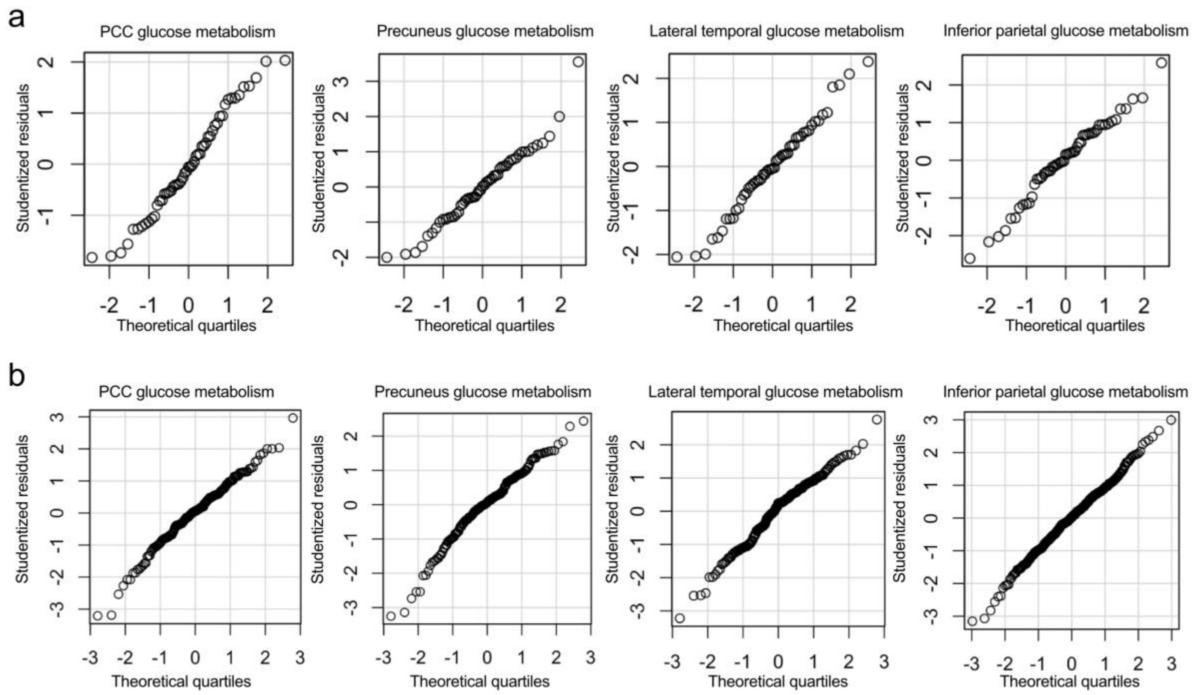

Supplementary Figure 3. Q-Q plots of the residuals for the models presented in Figure 1.

Q-Q plots of studentized residuals from the regression models presented in Figure 1 in (**a**) CN Aβ positive (see models in Figure 1d) and (**b**) MCI Aβ positive (see models in Figure 1e).

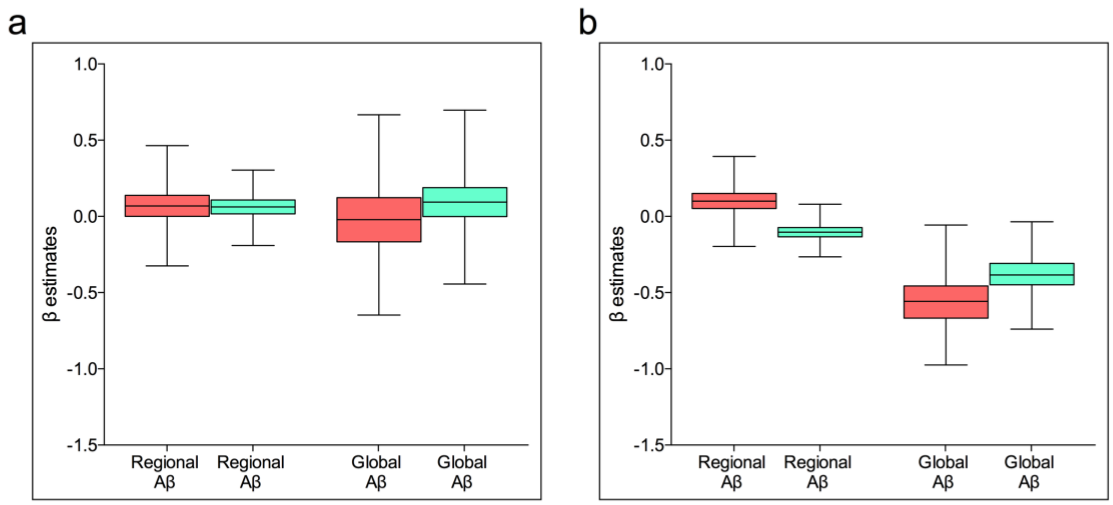

Supplementary Figure 4. Regional Aβ did not affect the association between global Aβ and metabolism. The figure shows the bootstrap-based estimates of the precuneus (regional) [^{18}F]florbetapir effect on precuneus [^{18}F]FDG with (red box) and without (green box) global [^{18}F]florbetapir effect in the models. Also, the bootstrap-based estimates of the global [^{18}F]florbetapir effect on precuneus [^{18}F]FDG with (red box) and without (green box) precuneus [^{18}F]florbetapir effect in (**a**) CN Aβ positive (n = 53) and (**b**) MCIs Aβ positive (n = 170). The models were also adjusted for age, gender, education, *APOE ε4* status, and p-tau. Resampling with replacement was iterated 10,000 times and sampling distributions of resulting estimates are presented as box plots. In the plots, the lower and upper boundaries show the 25th and 75th percentiles, respectively, the horizontal line indicates the median and the error bars show the minimum and maximum estimate values. The results suggest that the presence of regional [^{18}F]florbetapir did not cause significant instability in the model since the estimates that resulted from the models with and without regional [^{18}F]florbetapir effects showed similar association between global [^{18}F]florbetapir and regional [^{18}F]FDG. Results were similar for PCC, lateral temporal, and inferior parietal cortices.

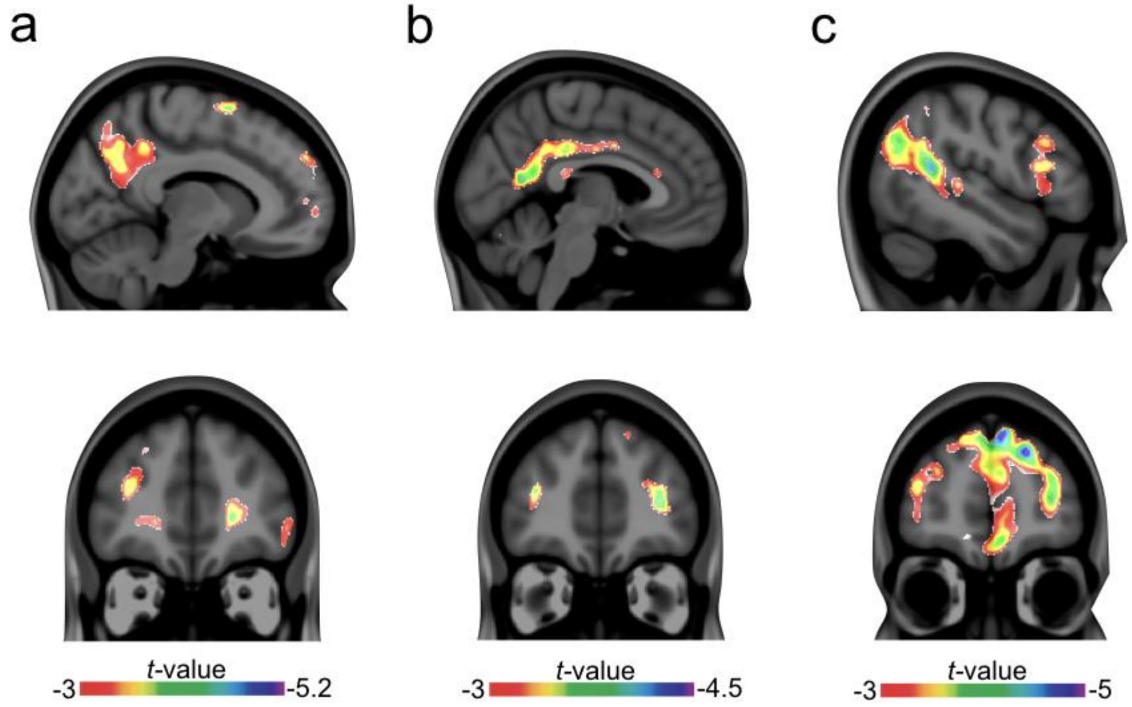

Supplementary Figure 5. The synergy of Aβ with overlapping hypometabolism on cognitive decline. Statistical parametric maps, FWER corrected at $P < 0.05$, show regions with a significant interactive effect between Aβ and glucose uptake on worsening in (**a**) Trail Making Test Part A, (**b**) Trail Making Test Part B, and (**c**) Boston Naming Test over 2 years in MCIs Aβ positive ($n = 170$). Aβ and glucose metabolism interaction did not significantly associate with changes in the Rey Auditory Verbal Learning Test 30-min delayed recall. There were no significant associations between the above-mentioned interaction and cognitive tests in CN Aβ positive ($n = 53$). Trail Making Test Part A assessed psychomotor speed processing, whereas Part B executive function. Boston Naming Test was used to test language and the Rey Auditory Verbal Learning Test assessed 30-min delayed recall memory.

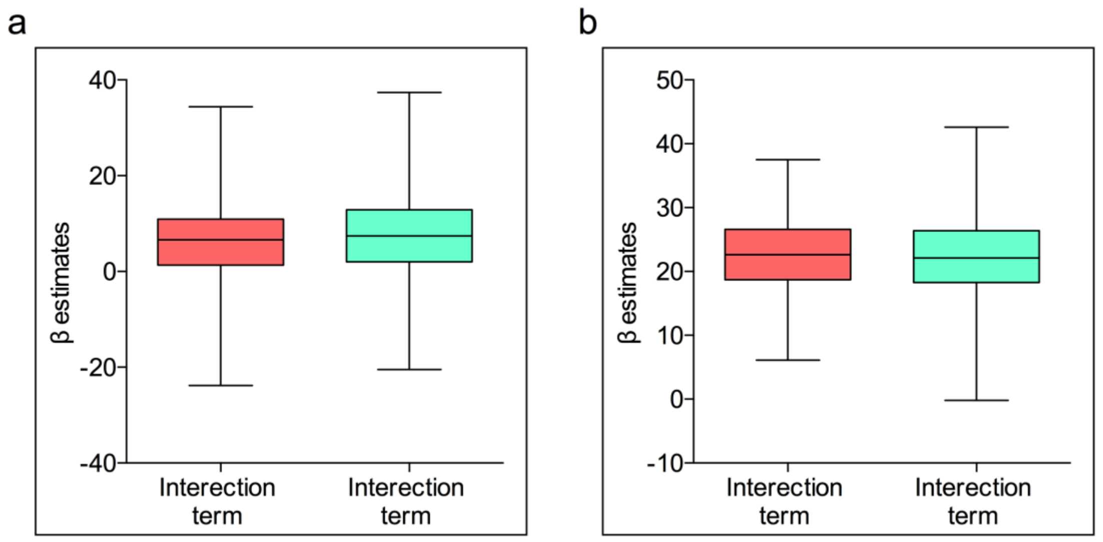

Supplementary Figure 6. Global Aβ values did not affect the stability of the local interaction between Aβ and hypometabolism. The figure shows the bootstrap-based estimates of the interactive effect of regional [^{18}F]florbetapir and [^{18}F]FDG on changes in cognition (MMSE) with (red box) and without (green box) global [^{18}F]florbetapir effect in (**a**) CN Aβ positive (n = 53) and (**b**) MCIs Aβ positive (n = 170) individuals. The models were also adjusted for age, gender, education, *APOE ε4* status, and p-tau. Resampling with replacement was iterated 10,000 times and sampling distributions of resulting estimates are presented as box plots. In the plots, the lower and upper boundaries show the 25th and 75th percentiles, respectively, the horizontal line indicates the median and the error bars show the minimum and maximum estimate values. Regional PET values used in the models were the mean of the average voxels presented in Figure 6a. The results suggest that the presence of global [^{18}F]florbetapir did not cause instability in the model since the estimates that resulted from the models with global [^{18}F]florbetapir effects were similar to the ones that resulted from the models without global [^{18}F]florbetapir effects.

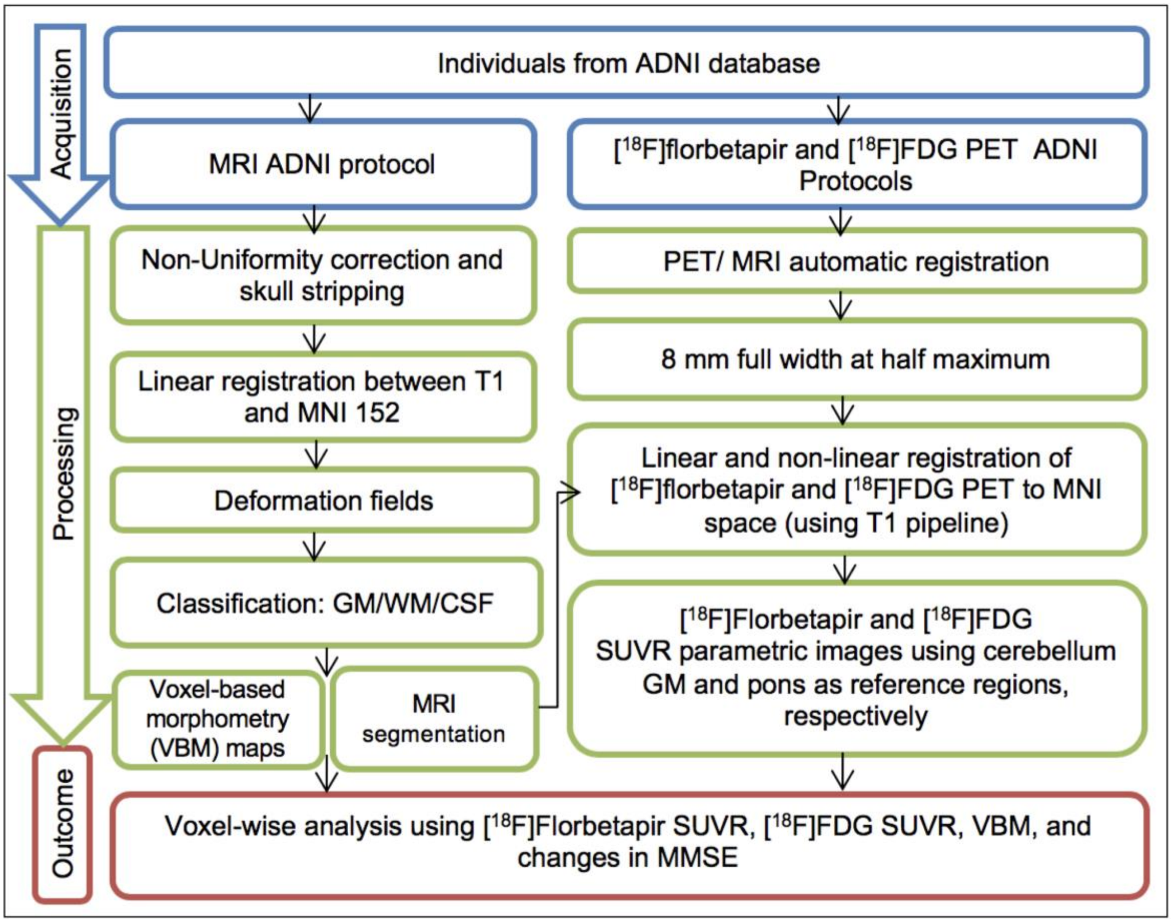

Supplementary Figure 7. Schematic representation of the human image pipeline.

GM = gray matter; WM = white matter.

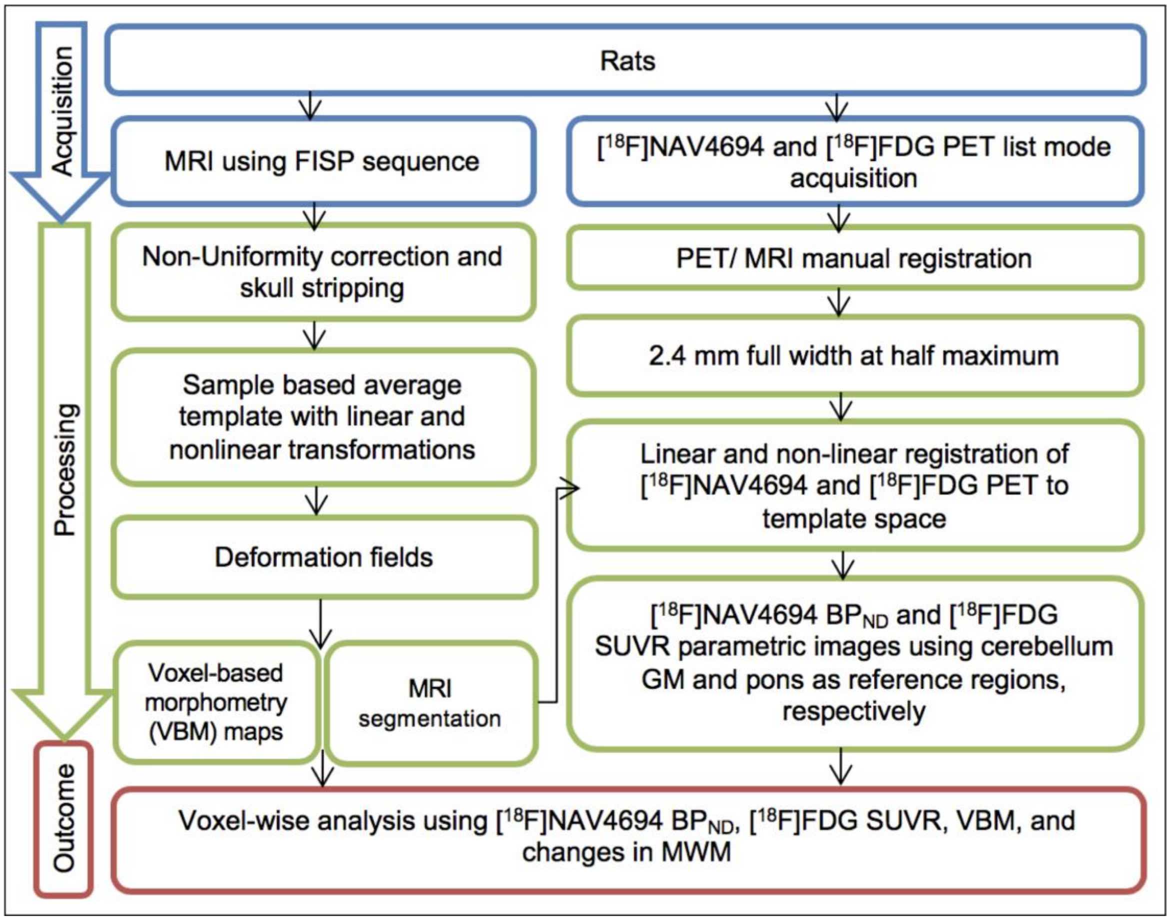

Supplementary Figure 8. Schematic representation of the rat image pipeline. FISP = Fast Imaging with Steady State Precession.
